# Injection preparation filtration and health concerns among indigenous people who inject methamphetamine

**DOI:** 10.3389/fpubh.2024.1390210

**Published:** 2024-06-12

**Authors:** Michael Anastario, Andrea Suarez, Olivia Williamson, Paula Firemoon, Elizabeth F. S. Roberts, Jarrett Barber

**Affiliations:** ^1^Center for Health Equity Research, Northern Arizona University, Flagstaff, AZ, United States; ^2^Robert Stempel College of Public Health & Social Work, Florida International University, Miami, FL, United States; ^3^Fort Peck Community College, Poplar, MT, United States; ^4^Department of Anthropology, University of Michigan, Ann Arbor, MI, United States

**Keywords:** methamphetamine, people who use injection drugs, injection preparations, filtration, health behaviors, American Indians

## Abstract

**Introduction:**

Injecting methamphetamine poses significant health risks, but little is known about how methamphetamine injectors filter their injection preparations and experience related health concerns.

**Methods:**

A chain-referral sample of Indigenous people who inject methamphetamine (*n* = 30) was recruited and semistructured interviews were conducted to collect information on filtration practices and health concerns.

**Results:**

Filtration of the injection preparation was described by 53% of injectors. Elevated levels of concern for kidney disease, cancer and heart disease were observed among those who filtered their preparations (ranging from 50 to 56.3%). Concern about liver disease was the most frequent concern among those who filtered their preparations (62.5%) and was elevated in comparison to those who did not use filters (7.1%). Grouped logistic regression revealed a positive association between filtration of the injection preparation and overall health concerns expressed by injectors, after adjusting for gender and age. The marginal posterior distribution of the adjusted odds ratio for filtration of the injection preparation had a posterior median = 35.7, and 95% HPD interval = (5.1, 512.4).

**Discussion:**

Results illustrate a positive relationship between filtration of the injection preparation and health concerns among Indigenous people who inject methamphetamine. This likely reflects the use of filtration to reduce harms, and further research is needed to understand the full scope of prevention that may be associated with filtration of methamphetamine injection preparations.

## 1 Introduction

Harm reduction strategies for people who use injection drugs have largely focused on reducing infectious disease and opioid-related risks, but less attention has been dedicated to understanding ways to reduce methamphetamine-centered risks. Injection methamphetamine use is a behavioral risk factor for multiple organ dysfunction and damage, psychosis, overdose and premature death. Understanding the health concerns and preventive behaviors employed by people who inject methamphetamine is important for developing methamphetamine injector-centered harm reduction interventions. Methamphetamine injectors’ filtration of their injection preparations may reflect an attempt by them to reduce harms, known or unknown, that may be associated with methamphetamine injection.

People who use injection drugs often use filters (e.g., cotton swabs, cigarette filters, Q-tips, paper towels) to draw the injection preparation into the syringe barrel (1, 2). This practice may reduce exposures to excipients, xenobiotics and other substances that may be present in unfiltered preparations (3–5). While filtration of the injection preparation may be a necessary aspect of the injection routine for people who inject pharmaceutical drugs containing excipients, water-soluble methamphetamine permits injectors to place the substance directly into the syringe barrel without subjecting the injection preparation to heat or filtration. It is unclear, however, what types of additional substances methamphetamine injectors may be exposed to, and whether filtration effectively reduces exposure to other agents. There is some evidence, for example, that disproportionate heavy metals exposures may occur for methamphetamine injectors due to the adulteration of illicit batches, (6–12) yet there are no studies that have examined whether filtration practices reduce such exposures or other potential toxicants beyond the harms associated with methamphetamine itself. This gap reflects a broader dearth of research on the injection routines of people who inject methamphetamine. While studies have examined filtration of the injection preparation in the context of oral pharmaceutical injections and general injection drug use, (13, 14) the filtration practices of methamphetamine injectors remain underexamined despite being of concern to methamphetamine injectors. The act of filtering methamphetamine injection preparations may reflect behavioral harm reduction strategies among methamphetamine injectors. Even though they may be unsubstantiated by current scientific evidence, behaviors that injectors experience to reduce harm may offer traction for future methamphetamine injector-centered harm reduction or behavior change interventions.

Little has been documented regarding methamphetamine injectors’ health concerns, but some studies have examined health concerns also among individuals who use methamphetamine by other routes of administration (non-injectors). In one study of 25 people who used methamphetamine in the state of Washington, 60% reported moderate to severe concerns regarding COVID-19 infection, with a considerable percentage expressing their intention to receive vaccination, prior to widespread vaccine availability (15). In studies of methamphetamine users (not necessarily injectors) who engage in sex while high (“chemsex”), participants have reported concerns regarding their own substance use, nonconsensual sex, transmitted infections and drug–drug interactions with HIV medication (16–20). While studies have illustrated that people who use methamphetamine experience notable health concerns, studies that have reported health concerns among methamphetamine users have not focused on the route of methamphetamine administration. It is important to document health concerns among methamphetamine injectors, particularly in relation to their injection routines, as methamphetamine injectors face intersecting stigmas and may be considered “beyond help” by some healthcare providers. It is a common assumption that methamphetamine use confounds injectors’ ability to experience risk, process health messages, and enact behavior change. Understanding methamphetamine injectors’ health concerns, particularly in relation to current injection routines, will be informative to the development of future harm reduction-motivated studies and interventions.

Finally, methamphetamine use is socially patterned. Over the first two decades of the 21st century, there has been a 50-fold increase in the methamphetamine mortality rate in the United States (21). Native Americans are the racial/ethnic group showing the highest and fastest growing death rates involving stimulants (22–25). Understanding harm-reducing behaviors implemented by people who inject methamphetamine can inform future interventions for reducing harm in similar populations where excess disease burdens are concentrated.

In this pilot research, we aimed to document the injection preparation filtration practices of people who inject methamphetamine in a Native American community where methamphetamine use is prevalent and to explore how these practices are related to concerns regarding health issues. Findings have implications for the development of methamphetamine injector-centered harm reduction science and strategies.

## 2 Methods

This study was conducted as part of a broader, community-engaged pilot research study that aimed to understand polysubstance use sequences among Indigenous people who use injection drugs. Life history calendar data from this study are reported elsewhere (26).

For this study, participants completed semistructured interviews that contained both close-ended and open-ended questions. In addition, observational field notes were taken during data collection and informed tailoring and refinement of interview questions, as this was a pilot data collection effort. The team’s approach was informed by a bioethnographic framework, which combines ethnographic and biostatistical data in symmetrical analyses to understand the relational and contingent phenomena of environment-body interactions, improving quantitative representations of health phenomena (27–29). As such, the measures below reflect questions included in the original draft of the interview instrument for all participants, while one measure reflects questions administered to only a subset of participants as it emerged during the study (Only qualitative data is reported for this measure obtained from a subset of individuals and is not included in the statistical analyses). Through interactions with people who use injection drugs during data collection, alternative potential filtration methods came to the study team’s attention through injectors’ emphasis on the act of filtration as a potential harm-reducing behavior. The current study reports on drug use filtration practices and health concerns among a sub-sample composed of methamphetamine injectors.

Participants were recruited through a chain-referral sampling strategy, which has been previously used by this research team to recruit Indigenous people who inject drugs at Fort Peck (30). Recruitment begins with selecting seed participants and proceeds with study participants recruiting others through word of mouth. This method is appropriate for hidden populations when members of the target population know one another and are densely interconnected (31, 32). The original pilot sample was generated using a chain-referral sampling strategy to identify participants. Inclusion criteria were being a federally recognized or associate tribal member and currently injecting drugs or who injected substances for ≥12 months during their lifespan. The study excluded individuals who were incarcerated and/or in police custody at the time of the study. Based on previous research and with additional input from community partners at Fort Peck, incentives for all participants included a $50 gift card. This amount was chosen to ensure that participants alloted the necessary time to complete the entire interview activity. Participants received a $50 gift card as an incentive. Participants completed semistructured interviews and life history calendars regarding their substance use. Of the 40 individuals who were recruited to the study, 75% (*n* = 30) identified as current methamphetamine injectors. As a small pilot study with a sample generated as part of a chain-referral sampling strategy, none of the participants declined to participate as they were generally aware of the study from prior participants. The subset of thirty methamphetamine injectors represents the analytic sample for the current study, and excludes individuals who did not report injecting methamphetamine.

This study was reviewed and approved by the Fort Peck Institutional Review Board (IRB), and the Florida International University IRB (Protocol Approval # IRB-21-0177). All participants provided verbal consent prior to participating in the interview. Semistructured interviews were conducted to collect information regarding injection filtration practices and health concerns.

### 2.1 Measures

#### 2.1.1 Injection preparation filtration

On semistructured interviews, participants were asked the open-ended question: “Now we are going to talk about the equipment, or “the works” that you use to inject. This includes syringes, spoons, cans, cookers, ties, cottons, filters, lighters, and the fluids you use to mix, cut, or cook down a substance. I’d like to start by asking you to walk me through a typical injection event. Please tell me about a typical process that you go through, and the materials that you use, to inject something into your body.” Open-ended responses were documented by the interviewer. Responses were then coded to evaluate whether any type of filtration device (e.g., cotton, Q-tip, cigarette butt, paper towel) was reported as being used as part of the injection routine. Coding indicated whether any of type of filtration was reported (filtration described/reported; filtration not described/not reported). If the respondent reported using any type of filtration in their routine, they were coded as filtering the injection preparation. A dichotomous variable was created to distinguish injectors who did not filter their injection preparations (0) from those who did filter their injection preparations (1).

#### 2.1.2 Health concerns

Participants were asked whether they were personally concerned about a series of health issues. Individual health issues were read aloud to each respondent. A checklist containing the following health issues was administered: Hepatitis C, HIV, Other infectious disease, opioid use disorder, any other addiction or dependency diagnosis, mental health diagnosis, diabetes, hypertension, heart disease, liver disease, cancer, or other illness. Concerns in the checklist are illustrated in Table 1. Dichotomous variables were created to represent concern for a given health issue (0 = not concerned, 1 = concerned about a given issues) for each of the 12 health concerns.

**Table 1:** Methamphetamine injection preparation filtration and health concerns among Indigenous injectors, *n* = 30.

Expressed personal concern regarding health issue	% Total	Does not filter injection preparation	Filters injection preparation
	(*n* = 30)	(*n* = 14)	(*n* = 16)
Cancer	33.3%	7.1%	56.3%
Kidney disease	26.7%	0.0%	50.0%
Abscesses	20.0%	0.0%	37.5%
Hepatitis C	40.0%	21.4%	56.3%
HIV	23.3%	7.1%	37.5%
Other infectious diseases	16.7%	7.1%	25.0%
Opioid use disorder	13.3%	0.0%	25.0%
Diabetes	36.7%	14.3%	56.3%
Hypertension	16.7%	0.0%	31.3%
Heart disease	33.3%	7.1%	56.3%
Liver disease	36.7%	7.1%	62.5%
Other illness	13.3%	7.1%	18.8%

#### 2.1.3 Acceptability of injection drug use syringe filters

Participants were shown pictures of injection drug use syringe filters (Sterifilt Plus ®) (33, 34). and asked if they would be willing to try these filters if they were provided at a future point in time. Any comments or utterances regarding the injection drug use syringe filter were recorded as open-ended responses. Only 23 of the 30 participants were asked questions about the Sterifilt filter as the idea for this intervention emerged from participants who were focused on the harm reducing benefits of filtering the injection preparation. Qualitative data from this question is included for context, but this measure was not included in any of the statistical analyses. The addition to the survey during the study was approved by the IRB and was articulated with a bioethnographic approach (27).

### 2.2 Data analysis

First, proportions of individual health concerns reported were examined relative to whether individuals filtered their injection preparations. The 12 health concern binary outcomes measured within each subject induce correlation among the outcomes within subjects. Accordingly, we used logistic regression to model the outcomes using subject random effects in the linear predictor to account for subjects who tend to have, for example, mostly low health concerns (mostly zeros) or mostly high health concerns (mostly ones); random subject effects were included for the linear predictor intercept and filtration effect, but not for the age and gender effects. Furthermore, each of these four effects (including intercept) were specified to vary with the 12 health concerns. Viewing the 12 health concern items as one set of many such possible sets of questions from which an “overall” concern for health could be gaged, each of the corresponding 12 effects---one set of 12 for each of the intercept, filtration, age and gender--- were viewed as arising from a “population” of possible health concern effects centered on an “overall” health effect, giving four overall health concern effects in total, including the intercept. Thus, overall effects on health concern were obtained for filtration, age and gender, plus an overall intercept, allowing overall health concern odds and odds ratios to be inferred in a typical fashion. The resulting hierarchical nature of the model allowed 12 different health concern effects to be distinguished, if the data permit, otherwise shrinking each of the 12 effects toward their respective overall population center when the data do not contain enough information to distinguish among the 12 health concerns. The overall effects are arguably of main interest, particularly the overall effect of filtration on health concern. The model was implemented within a Bayesian framework with relatively vague priors; see the Supplementary material for model details and Stan code.

## 3 Results

Sixteen (53.3%) of the participants were male, and 14 (46.7%) were female (Table 2). The majority of participants were 30–40 years of age (46.7%) or 40–50 years of age (36.7%). The majority were single (56.7%). Twenty-eight participants (93.3%) were federally recognized tribal members, and two (6.7%) were associate tribal members. The majority of participants identified as Sioux (70.0%), followed by both Assiniboine and Sioux (16.7%), other (10.0%) and Assiniboine (3.3%) (Table 2).

**Table 2:** Demographic characteristics of people who inject methamphetamine, *n* = 30.

Characteristic	*N* (%)
Gender identity	
Male	16 (53.3%)
Female	14 (46.7%)
Age	
20–30	3 (10.0%)
30–40	14 (46.7%)
40–50	11 (36.7%)
50–65	2 (6.7%)
Relationship status	
Single	17 (56.7%)
Divorced/separated	2 (6.7%)
Married	3 (10.0%)
Unmarried w/partner	7 (23.3%)
Widowed/widower	1 (3.3%)
Tribal recognition status	
Registered member of a federally recognized tribe	28 (93.3%)
Associate tribal member	2 (6.7%)
Tribal affiliaton	
Assiniboine	1 (3.3%)
Sioux	21 (70.0%)
Both Assiniboine and Sioux	5 (16.7%)
Other*	3 (10.0%)

*Includes Chippewa, Innuit, and Potawatomi.

Among participants, 53% (*n* = 16) reported filtering their injection preparations. While individuals who filtered their injection preparations reported more health concerns for each health condition examined, the largest between-group differences occurred for: liver disease (62.5% of those who filtered injection preparations reported concern versus 7.1% of those who did not filter their injection preparations), kidney disease (50% vs. 0%), cancer (56.3% vs. 7.1%), and heart disease (56.3% vs. 7.1%) (Table 1).

For the Bayesian analysis, the resulting marginal posterior distribution for the terms in the model are depicted in the Supplementary materials. The Bayesian hierarchical model demonstrated good convergence for the estimated effects of filtration, gender, and age on individual health concerns, as evidenced by potential scale reduction factor values close to 1. The marginal posterior distribution of the adjusted odds ratio for filtration of the injection preparation had a posterior median = 35.7, and 95% HPD interval = (5.1, 512.4). The posterior medians for the effects of age and gender did not suggest appreciable positive or negative effects on health concerns (Table 3).

**Table 3:** Posterior estimates and 95% highest posterior density intervals for the overall effects of filtration, age, and gender on health concerns.*

	Posterior median	95% Highest posterior density interval
Intercept	0.0	0.0–0.1
Filters injection preparation	35.7	5.1–512.4
Age	0.6	0.0–43.4
Gender (males are referent)	1.1	0.1–8.1

*Baseline odds of concern (beta_k_, *k* = 0) and odds ratios for filtration, age and gender (*k* = 1, 2, 3), for the baseline of a middle-aged (44.5 year) male who does not use filtration.

Among participants who reported filtering their injection preparations, 93.3% reported being willing to try the filters if they were provided. Participants generally expressed accepting attitudes toward the introduction of syringe filters, with many indicating a willingness to try them (Table 4). Common themes presented by participants included perceived benefits over traditional methods, such as cotton, due to potential reductions in bacterial infections and removal of insoluble particles. For example, one participant described how: “That would be a good thing. Cottons collect dust. I talk with my boyfriend about all the chemicals in dope killing bacteria, but we do not know and who knows what else is in there.” Another participant described that “I would like to try it because it would probably be better than a cotton. But I still prefer the “shake and bake” or “shake and wake” method because it does not leave works behind. It’s cleaner.” However, some skepticism was noted, with preferences for existing practices and concerns about the newness and appearance of the filters.

**Table 4:** Open-ended comments regarding injection drug use syringe filters provided by participants.*

A lot of people would use those. I know for a fact. I do not know, I’d have to try it out and let you know. Can you call me and let me know where to get those? I would like to try it because it would probably be better than a cotton. But I still prefer the “shake and bake” or “shake and wake” method because it does not leave works behind. It’s cleaner. I would try it I’ll try it It do not look right It is something new. It would be cool because I do not always wash my hands or disinfect my hands. That would be pretty neat. I’d try it just to try it No because I do not use works I know I got bacterial infections from dirty needles or spoons, so this looks good. I think others would be open to it. I’d tell them to use it. That would be a good thing. Cottons collect dust. I talk with my boyfriend about all the chemicals in dope killing bacteria, but we do not know and who knows what else is in there. Wow. It looks like a good idea. Yeah, but I always use cotton

*Nine participants did not provide a comment.

## 4 Discussion

In this study of Indigenous people who inject methamphetamine, we found that filtration of the injection preparation was associated with a greater number of health concerns. Results suggest that injectors who routinely filter their injection preparations experience a greater number of health concerns, and may reflect an attempt by injectors to engage in potentially less toxic modalities of injection methamphetamine use. Results have implications for future studies that will promote the science of harm reduction.

Filtration of methamphetamine injection preparations may be a valid method for reducing exposure to additional substances, which requires further investigation. The efficacy of quotidian versus “gold standard” injection drug use syringe filters (IDUSFs) for reducing exposure to additional substances within illicit methamphetamine samples has not yet been evaluated among people who use injection drugs. IDUSFs have been shown to remove particles of pharmaceutical medications, including buprenorphine tables (approximately 85% of particles between 1 and 5 mm and 97% of particles between 5 and 18 mm), and particles of methylphenidate (approximately two-thirds of particles between 1 and 5 mm and 95% of particles between 5 and 18 mm), which may otherwise contribute to potential harms such as pulmonary emboli among people who use injection drugs (13). However, the efficacy of IDUSFs in removing particles from methamphetamine injection preparations has not been evaluated in the extant literature. While IDUSFs have been shown to remove bacterial contamination more successfully in comparison to routine filters, (14) there is still a need to understand what routine filtration practices and IDUSFs remove from injection preparations that specifically contain methamphetamine. The late 20th century proliferation of illicit methamphetamine drug laboratories in the US was met with calls for environmental assessments to sample laboratory sites for inorganic and organic compounds as well as heavy metals (35). Methods of illicit methamphetamine production include using ephedrine, pseudoephedrine, or 1-phenyl-2-propanone (P2P) as precursors (36–39). There is some evidence that disproportionate lead and mercury exposures may occur for methamphetamine injectors due to the adulteration of batches (6, 8, 9, 12, 40–42). However, exposures have not been evaluated in relation to filtration of the injection preparation, and further research on this topic is warranted, particularly as the authors are unaware of any studies that evaluate methods for removing soluble metals from methamphetamine injection preparations.

Further research on filtration practices and exposure to potential additional substances among people who inject methamphetamine is important to consider for harm reduction interventions that may consider the use of IDUSFs with methamphetamine injectors. Among people who use injection drugs, filtration of the injection preparation is one component of what are often intimate and routinized aspects of drug injection routines implicating drug injection equipment (43, 44). In one qualitative study of Norwegian people who use injection drugs, participants described injection routines as bringing joy, peace, and excitement; experiences that highlight the emotional and sensory aspects of the experience (45). Many people who use injection drugs view injection routines as a central aspect of injecting and can develop a dependence on the routine itself, with some making incremental changes to their injection routines over time that may be hard to change (46, 47). Generating more evidence surrounding a specific component of the injection routine – filtration – could be a cornerstone for implementing methamphetamine-injector centered harm reduction strategies.

This study has several strengths and weaknesses. The small sample size reflects a broader concern regarding statistical power when conducting research within heterogeneous tribal communities. We used Bayesian modeling to address small sample size concerns and uncertainty regarding the parameters. It is admittedly difficult to conduct research on tribal lands with health disparity populations that are both stigmatized and sensitive to interrogation. Despite having a small sample size, the study illustrates how people who inject methamphetamine take active steps to decrease harms associated with injection drug use by filtering the injection preparation, and it shows appreciable interest and acceptability for using injection drug use syringe filters in future harm reduction efforts in this population. The $50 incentive was designed to respectfully compensate participants for their time and effort in completing the study. However, this amount might inadvertently introduce recruitment biases. Specifically, it may not be enough to attract working individuals for whom the financial compensation does not justify the time required to participate. Only 23 of the 30 participants were asked questions about the acceptability of injection drug use syringe filters, and the qualitative data reported in Table 4 do not represent all participants. Finally, the addition of a survey item linked to observational data during the course of the pilot study reflects the study being methodologically informed by a bioethnographic approach and an ethical imperative to recognize “participants as knowers” (48, 49). This study received tribal IRB approval and successfully demonstrated engagement with Indigenous methamphetamine injectors regarding intimate and sensitive aspects regarding their illicit substance use, and will serve as the foundation for future research aimed at understanding how filtration practices may reduce exposure to environmental toxicants among Indigenous people who inject methamphetamine.

## 5 Conclusion

This study demonstrates an association between filtration of the injection preparation and a greater number of health concerns among Indigenous people who inject methamphetamine. This association encourages research into the efficacy of filtration practices for removing additional substances from illicit methamphetamine and emphasizes one behavior that may be a fruitful point of departure for methamphetamine injector-centered harm reduction strategies. Future research should explore the efficacy of filters in reducing potential contaminants and other substances in methamphetamine injection preparations as well as investigate the broader behavioral and psychological aspects underlying these harm reduction practices.

## Data availability statement

The datasets presented in this article are not readily available because Access to the dataset must be granted only after having received approval from the Fort Peck Tribal Institutional Review Board. Requests to access the datasets should be directed to michael.anastario@nau.edu.

## Ethics statement

The studies involving humans were approved by Fort Peck Tribal Institutional Review Board; Florida International University Institutional Review Board. The studies were conducted in accordance with the local legislation and institutional requirements. The ethics committee/institutional review board waived the requirement of written informed consent for participation from the participants or the participants’ legal guardians/next of kin because Written consent was waived to reduce potential breaches of confidentiality, verbal consent was obtained from all participants.

## Author contributions

MA: Conceptualization, Methodology, Project administration, Writing-original draft. AS: Writing-review & editing. OW: Writing-review & editing. PF: Writing-review & editing. ER: Writing-review & editing. JB: Methodology, Writing-original draft, Writing-review & editing.
