# Supplementary material for: Injection preparation filtration and health concerns among indigenous people who inject methamphetamine
Source: Front Public Health. 2024 Jun 12;12:1390210. doi: 10.3389/fpubh.2024.1390210 (PMC11201683; doi:10.3389/fpubh.2024.1390210)
Supplement: Supplementary file 1 [file Data_Sheet_1.PDF]

# Chapter 1

# Effects of Filtration on Health Concerns

## 1.1 Stage One Model: Conditionally Independent Health Concern Outcomes

Consider binary outcomes, $Y_{ij}$, to indicate (0/1) health concern $j$, $j = 1, \ldots, J = 12$, for subject $i$, $i = 1, \ldots, n = 30$, with probabilities of a concern $p_{ij} = Pr(Y_{ij} = 1)$, and probabilities of no concern, $(1 - p_{ij}) = Pr(Y_{ij} = 0)$.

As the $J$ health concern outcomes are measured from each subject, it is likely that these outcomes are positively correlated within each subject as, e.g., one subject may tend to have high probabilities of concerns (and positive (1) outcomes) across all $J$ concerns while another subject may tend to have low probabilities (and negative (0) outcomes). We account for correlation amongst outcomes via their probabilities, as detailed below.

Conditional on the probabilities of concern outcomes, we model the concern outcomes as independent Bernoullis,

$$Y_{ij} \sim \text{Bern}(p_{ij}),$$

and we model the log odds of concern probabilities as

$$\text{logit}(p_{ij}) = \beta_{0ij} + \beta_{1ij} x_{i1} + \beta_{2j} x_{i2} + \beta_{3j} x_{i3}$$

where $x_{i1}$ indicates (0/1) subject $i$ to filter injection preparation, $x_{i2}$ indicates subject gender, $x_{i3}$ subject age, and the $\beta$ effects are specified below.

## 1.2 Stage Two Model: Subject and Health Concern Outcome Effects

As introduced, above, we account for correlation amongst the $J$ health concern outcomes (and amongst their probabilities) within each subject by modeling effects $\beta_{kij}$ in the following manner. Let

$$\beta_{kij} = \beta_{ki} + \beta_{kj},$$

where $\beta_{ki}$ is an effect for subject $i$ and $\beta_{kj}$ is an effect for health concern outcome $j$, each case being of effect type $k$

### 1.2.1 Independent Random Subject Effects

We envision subject effects to arise independently from a subject effect population

$$\beta_{ki} \sim N(0, \sigma^2_{\beta_{sk}})$$

Thus, the $i$th subject's random effects, $\beta_{0i}$ and $\beta_{1i}$, are shared among $j = 1, \ldots, J$ health outcome within subject $i$, thus inducing correlation among the $J$ health outcomes within subjects as mentioned previously. Random subject effects seem intuitive for the baselines (linear predictor intercepts $k = 0$) and filtration effects ($k = 1$), but less so for the sex and age effects ($k = 2, 3$), which preliminary analyses (not shown) suggest do not need such random subject effects, i.e., preliminary analyses (not shown) indicated their would-be variance components $\sigma^2_{\beta_{sk}}$, $k = 2, 3$, are relatively small, near zero. Thus, $\beta_{2i} = \beta_{3i} = 0$ in our analyses reported here, and there are no corresponding variance components.

### 1.2.2 Independent Random Health Concern Outcome Effects

We envision the $J$ health concern outcome effects $\beta_{kj}$ as arising from conceptual, latent populations of such effects from which we could have originated other, particular health concern outcome effects for practically same purpose of assessing essentially the same overall sort of health outcome concern.

Thus, we allow $J$ effects across outcomes to vary about a population mean—overall—effect, $\beta_k$,

$$\beta_{kj} \sim N(\beta_{ck}, \sigma_{ck}^2)$$

$k = 0, \ldots, 3$. Equivalently, we may write

$$\beta_{kj} = \beta_{ck} + b_{kj},$$

with

$$b_{kj} \sim N(0, \sigma_{ck}^2).$$

## 1.3 Stage Three Priors

We complete our model specification with prior distributions on the subject effects population variance components $\sigma_{sk}^2$, $k = 0, 1$ (folded $t$ distributions), the overall health concern outcome population mean effects, $\beta_k$, $k = 0, \ldots, 3$ (normal distributions), and the overall health concern outcome population effects variance components, $\sigma_{ck}^2$, $k = 0, \ldots, 3$ (folded $t$ distributions). See the Stan Implementation for details on these priors.

# Chapter 2

# Stan Implementation

As discussed, the code implements the stages of the model discussed above. The $J = 12$ health concern outcome variables for each of the $n = 30$ subjects are specified with a Bernoulli model in stage 1.

The subject intercept ($\beta_{0i}$) and filteration ($\beta_{1i}$) random effects are specified as normal with means 0 and variance components ($\sigma^2_{sk}$, $k = 0, 1$) specified as folded-t's via parameter expansion (see Andrew Gelman (2004), Parameterization and Bayesian Modeling, Journal of the American Statistical Association 99(466):537-545, DOI 10.1198/016214504000000458; and see Andrew Gelman (2006), Prior distributions for variance parameters in hierarchical models, Bayesian Analysis 1(3):515–533).

The $p = 4$ overall effects ($\beta_{ck}$, $k = 0, \ldots, 3$) are given relatively vague normal priors, especially considering the log odds scale of these effects; $N(0, 10)$ where 10 is a standard deviation, not variance. Again, these $p = 4$ overall effects serve as the means of populations from which 12 concern effects arise. Each of these 4 concern populations also has its own variance component ($\sigma^2_{ck}$) assigned folded-t prior distributions implemented via parameter expansion to avoid sampling problems associated with small variance components and associated small effects.

The folded-t priors for the population variance components allows them to be close to zero, without the aforementioned sampling problems, while having thick enough tails for larger components to be estimated from the data, which we do not expect a priori for such variance components of effects on the log odds scale, especially because all covariates operate within a range of 1 and we would not expect large changes in effects on the log odds scale as covariates cover their entire range; age is normalized to the range of [-0.5,

0.5] so that the baseline (log) odds is associated with a non-filtering male with a mid-range age (44.5 yrs).

## 2.1 Stan Code

```
> writeLines(readLines("./Stan/healthconcerns.stan"))

functions {
  // none
}

data {
  int<lower=1> n;                         // # subjects
  int<lower=1> J;                         // # health concerns (J in text)
  int<lower=1> k;                         // # covariates (not effect/x subscript as in text
  int<lower=1> p;                         // # overall parameters (k+1)
  int<lower=1, upper=p> d;                // # of random coefs (in b below)
  array[n,J] int<lower=0, upper=1> y; // # outcome of sub i, concern j
  array[n,p] real x;                      // covariate "matrix" including 1's column
}

transformed data{
  // none
}

parameters{
  array[p] real beta_ovall;           // overall effects
  array[p] real cw;                   // for parameter expansion (pe) of concern effects
  array[p,J] real ceps;               // for pe of concern effects
  array[p] real<lower=0> sigmaceps2; // for pe of concern effects
  array[d] real sw;                   // for pe of subject effects
  array[d,n] real seps;               // for pe of subject effects
  array[d] real<lower=0> sigmaseps2; // for pe of subject effects

}

transformed parameters{
  array[p] real<lower=0> sigmac; // concern effects sd comps (sqrts of var. comps.)
```

```
  array[d] real<lower=0> sigmas; // subject effects sd comps (sqrts of var. comps.)
  array[p,J] real beta_concerns; // concern effects
  array[d,n] real beta_subjects; // subject effects
  for(kk in 1:p) {sigmac[kk] = abs(cw[kk]) * sqrt(sigmaceps2[kk]);
    for(jj in 1:J) {beta_concerns[kk][jj] = beta_ovall[kk] +
cw[kk] * ceps[kk][jj];}}
  for(kk in 1:d) {sigmas[kk] = abs(sw[kk]) * sqrt(sigmaseps2[kk]);
    for(ii in 1:n) {beta_subjects[kk][ii] = sw[kk] * seps[kk][ii];}}

}

model{

  array[p,n] real ball;
  array[p,n,J] real beta;
  array[n,J] real eta;

  // All additive random subject and random concern effects, now
  // including zero subject random effects for age and gender
  for(i in 1:n){    // subject id
    for(kk in 1:p){ // effect type (k=0,1,2,3 in text)
      if(kk <= d) ball[kk][i] = beta_subjects[kk][i];
      else ball[kk][i] = 0.0;
    }
  }

  // Linear predictor and data model specifiction
  for(i in 1:n){       // subject id
    for(j in 1:J){     // concern id
      eta[i][j] = 0.0;
      for(kk in 1:p){  // effect type (0,1,2,3 in text)
beta[kk][i][j] = ball[kk][i] + beta_concerns[kk][j];
eta[i][j] += beta[kk][i][j]*x[i][kk];
      }
      y[i][j] ~ bernoulli_logit(eta[i][j]);
    }
  }

  // Concern level priors
```

```
  for(kk in 1:p){
    for(j in 1:J) {
      ceps[kk][j] ~ normal(0,sqrt(sigmaceps2[kk])); // for pe of concern effects
    }
    beta_ovall[kk] ~ normal(0, 10); // concerns means priors
    cw[kk] ~ normal(0,1);           // for pe of concern effects
    sigmaceps2[kk] ~  scaled_inv_chi_square(0.1,1); // for pe of concern effects
  }

  // subject level priors
  for(i in 1:n){
    seps[1][i] ~ normal(0, sqrt(sigmaseps2[1])); // for pe of subject effects
    seps[2][i] ~ normal(0, sqrt(sigmaseps2[2])); // for pe of subject effects
  }
  sigmaseps2[1] ~ scaled_inv_chi_square(0.1,1); // for pe of subject effects
  sigmaseps2[2] ~ scaled_inv_chi_square(0.1,1); // for pe of subejct effects
  sw[1] ~ normal(0,1); // for pe of subject effects
  sw[2] ~ normal(0,1); // for pe of subject effects
}
```

## 2.2 Translate, Compile and Link

```
> ## not evaluated when knitting
> library(cmdstanr)
> file <- file.path("./Stan/healthconcerns.stan")
> mod <- cmdstan_model(file)
```

## 2.3 Data

```
> injectors.df<- as.data.frame(
+     readxl::read_xls(path="injectors.xls",
+                      sheet=1))
> ## str(injectors.df)
```

```
>
> ## Normalize (scale) age to [-0.5, 0.5]
> scage<- with(injectors.df, (age - min(age))/diff(range(age)) - 0.5)
>
> ## (resulting baseline reference age:)
> min(injectors.df$age) + 0.5 * diff(range(injectors.df$age))

[1] 44.5

> ## Regression matrix with normalized (scaled) age:
> x<- cbind(1,injectors.df$filters,scage,injectors.df$female)
>
> ## To pass to Stan:
> injectors.list<- list(
+     n = 30,
+     J = 12,
+     k = 3,
+     p = 4,
+     d = 2,
+     y = as.matrix(injectors.df[,1:12], dim=dim(injectors.df[,1:12])),
+     x = as.matrix(x))
>
> rm(scage, x) ## clean up
```

## 2.4 Sampling

This should be fairly self-explanatory. Note, Stan conveniently generates starting values of all parameters in all chains without any apparent problems.

```
> ## not evaluated when knitting
> fit <- mod$sample(
+                 data = injectors.list,
+                 iter_sampling = 10000,
+                 iter_warmup = 20000,
+                 save_warmup = TRUE,
+                 seed = 123,
```

```
+                chains = 4,
+                parallel_chains = 4,
+                refresh = 500
+            )
```

## 2.5 Convergence Diagnostics

While the cmdstanr R package typically incorporates the latest changes to the Stan language in a timely manner, cmdstanr has relatively limited high-level functionality for diagnosing convergence of chains to the posterior or for summarizing the posterior. For these purposes, we may use rstan or coda, the latter used here after converting posterior samples to the appropriate object type for use by coda; the fit object is suited for analysis by cmdstanr, the stanfit object is suited for analysis by rstan, the fit.mcmc.list object is suited for analysis by coda and the fit.array object may be best suited for analysis using more typical R functions.

```
> ## not evaluated when knitting
> ## Create rstan stanfit object on the way to a coda mcmc list object.
> stanfit <- rstan::read_stan_csv(fit$output_files())
> fit.array<- rstan::extract(stanfit, permuted=FALSE, inc_warmup=TRUE)
> dim(fit.array)
> nchains<- dim(fit.array)[2]
> fit.mcmc.list<- vector("list", nchains)
> for(chain in 1:nchains){
+     fit.mcmc.list[[chain]]<- coda::as.mcmc(fit.array[,chain,])
+     }
> fit.mcmc<- coda::as.mcmc.list(fit.mcmc.list)
> save(list=c("fit", "stanfit","fit.array", "fit.mcmc.list","fit.mcmc"),
+      file="stanfit.RData")
```

Only identifiable parameters are retained to assess convergence; quantities used exclusively to implement parameter expansion are excluded from both posterior convergence analysis and summaries. Theory does not support sample convergence to the posterior during Stan's warmup period, so

these samples should be excluded before assessing convergence and summarizing. Both graphical and quantitative measures indicate convergence to the posterior.

```
> load(file="stanfit.RData")
> library(coda)
> names<- varnames(fit.mcmc)[c(1:4,125:239)] ## exclude par expansion quantities
```

```
> ## Graphical convergence diagnostics (not run to reduce file size)
> plot(fit.mcmc[,names], ask=FALSE) ## visually ignore iterations before 10001
```

```
> ## psrf convergence diagnostics
> gelman.diag(fit.wind.mcmc<- window(fit.mcmc[,names], start=10001))

Potential scale reduction factors:

                    Point est. Upper C.I.
beta_ovall[1]             1.00       1.01
beta_ovall[2]             1.00       1.00
beta_ovall[3]             1.00       1.00
beta_ovall[4]             1.00       1.00
sigmac[1]                 1.00       1.00
sigmac[2]                 1.00       1.00
sigmac[3]                 1.00       1.00
sigmac[4]                 1.00       1.00
sigmas[1]                 1.00       1.00
sigmas[2]                 1.01       1.01
beta_concerns[1,1]        1.00       1.01
beta_concerns[2,1]        1.00       1.00
beta_concerns[3,1]        1.00       1.00
beta_concerns[4,1]        1.00       1.00
beta_concerns[1,2]        1.00       1.00
beta_concerns[2,2]        1.00       1.00
beta_concerns[3,2]        1.00       1.00
beta_concerns[4,2]        1.00       1.00
beta_concerns[1,3]        1.00       1.01
beta_concerns[2,3]        1.00       1.00
beta_concerns[3,3]        1.00       1.00
```

```
beta_concerns[4,3]        1.00       1.00
beta_concerns[1,4]        1.00       1.01
beta_concerns[2,4]        1.00       1.00
beta_concerns[3,4]        1.00       1.00
beta_concerns[4,4]        1.00       1.00
beta_concerns[1,5]        1.00       1.01
beta_concerns[2,5]        1.00       1.00
beta_concerns[3,5]        1.00       1.00
beta_concerns[4,5]        1.00       1.00
beta_concerns[1,6]        1.00       1.00
beta_concerns[2,6]        1.00       1.00
beta_concerns[3,6]        1.00       1.00
beta_concerns[4,6]        1.00       1.00
beta_concerns[1,7]        1.00       1.00
beta_concerns[2,7]        1.00       1.00
beta_concerns[3,7]        1.00       1.00
beta_concerns[4,7]        1.00       1.00
beta_concerns[1,8]        1.00       1.00
beta_concerns[2,8]        1.00       1.00
beta_concerns[3,8]        1.00       1.00
beta_concerns[4,8]        1.00       1.00
beta_concerns[1,9]        1.00       1.01
beta_concerns[2,9]        1.00       1.00
beta_concerns[3,9]        1.00       1.00
beta_concerns[4,9]        1.00       1.00
beta_concerns[1,10]       1.00       1.01
beta_concerns[2,10]       1.00       1.00
beta_concerns[3,10]       1.00       1.00
beta_concerns[4,10]       1.00       1.00
beta_concerns[1,11]       1.00       1.01
beta_concerns[2,11]       1.00       1.00
beta_concerns[3,11]       1.00       1.00
beta_concerns[4,11]       1.00       1.00
beta_concerns[1,12]       1.00       1.01
beta_concerns[2,12]       1.00       1.00
beta_concerns[3,12]       1.00       1.00
beta_concerns[4,12]       1.00       1.00
beta_subjects[1,1]        1.00       1.00
beta_subjects[2,1]        1.00       1.00
```

```
beta_subjects[1,2]         1.00       1.00
beta_subjects[2,2]         1.00       1.00
beta_subjects[1,3]         1.00       1.00
beta_subjects[2,3]         1.01       1.02
beta_subjects[1,4]         1.00       1.00
beta_subjects[2,4]         1.00       1.01
beta_subjects[1,5]         1.00       1.00
beta_subjects[2,5]         1.00       1.00
beta_subjects[1,6]         1.00       1.00
beta_subjects[2,6]         1.01       1.01
beta_subjects[1,7]         1.00       1.00
beta_subjects[2,7]         1.01       1.02
beta_subjects[1,8]         1.00       1.00
beta_subjects[2,8]         1.00       1.00
beta_subjects[1,9]         1.00       1.00
beta_subjects[2,9]         1.00       1.01
beta_subjects[1,10]        1.00       1.00
beta_subjects[2,10]        1.00       1.00
beta_subjects[1,11]        1.00       1.00
beta_subjects[2,11]        1.00       1.00
beta_subjects[1,12]        1.00       1.00
beta_subjects[2,12]        1.00       1.00
beta_subjects[1,13]        1.00       1.00
beta_subjects[2,13]        1.00       1.00
beta_subjects[1,14]        1.00       1.00
beta_subjects[2,14]        1.01       1.01
beta_subjects[1,15]        1.00       1.00
beta_subjects[2,15]        1.00       1.01
beta_subjects[1,16]        1.00       1.00
beta_subjects[2,16]        1.00       1.00
beta_subjects[1,17]        1.00       1.00
beta_subjects[2,17]        1.00       1.01
beta_subjects[1,18]        1.00       1.00
beta_subjects[2,18]        1.00       1.00
beta_subjects[1,19]        1.00       1.00
beta_subjects[2,19]        1.00       1.01
beta_subjects[1,20]        1.00       1.00
beta_subjects[2,20]        1.00       1.00
beta_subjects[1,21]        1.00       1.00
```

```
beta_subjects[2,21]        1.00       1.00
beta_subjects[1,22]        1.00       1.00
beta_subjects[2,22]        1.01       1.01
beta_subjects[1,23]        1.00       1.00
beta_subjects[2,23]        1.00       1.00
beta_subjects[1,24]        1.00       1.00
beta_subjects[2,24]        1.00       1.01
beta_subjects[1,25]        1.00       1.00
beta_subjects[2,25]        1.00       1.00
beta_subjects[1,26]        1.00       1.00
beta_subjects[2,26]        1.01       1.01
beta_subjects[1,27]        1.00       1.00
beta_subjects[2,27]        1.00       1.01
beta_subjects[1,28]        1.00       1.01
beta_subjects[2,28]        1.00       1.00
beta_subjects[1,29]        1.00       1.00
beta_subjects[2,29]        1.00       1.01
beta_subjects[1,30]        1.00       1.00
beta_subjects[2,30]        1.00       1.00
lp__                       1.00       1.01

Multivariate psrf

1.02
```

## 2.6 Posterior Summary

Here are typical posterior summaries provided by `coda`.

```
> options(width=80)
> summary(fit.wind.mcmc)


Iterations = 10001:30000
Thinning interval = 1
Number of chains = 4
Sample size per chain = 20000
```

```
1. Empirical mean and standard deviation for each variable,
   plus standard error of the mean:

                        Mean     SD  Naive SE Time-series SE
beta_ovall[1]      -4.351e+00 1.0935 0.0038662       0.013007
beta_ovall[2]       3.670e+00 1.1658 0.0041217       0.009425
beta_ovall[3]      -5.301e-01 2.1401 0.0075663       0.018172
beta_ovall[4]       6.425e-02 1.0335 0.0036540       0.008468
sigmac[1]           6.281e-01 0.3641 0.0012873       0.002471
sigmac[2]           4.478e-01 0.3467 0.0012259       0.002428
sigmac[3]           4.757e-01 0.4499 0.0015907       0.005008
sigmac[4]           3.122e-01 0.2688 0.0009503       0.002616
sigmas[1]           2.085e+00 0.6440 0.0022769       0.006068
sigmas[2]           7.319e-01 0.6873 0.0024301       0.012739
beta_concerns[1,1] -4.409e+00 1.1592 0.0040983       0.013129
beta_concerns[2,1]  3.602e+00 1.2172 0.0043033       0.009045
beta_concerns[3,1] -4.713e-01 2.2082 0.0078071       0.019890
beta_concerns[4,1] -6.086e-02 1.0910 0.0038574       0.009112
beta_concerns[1,2] -3.726e+00 1.1391 0.0040274       0.011883
beta_concerns[2,2]  3.755e+00 1.2076 0.0042696       0.009071
beta_concerns[3,2] -5.318e-01 2.1910 0.0077465       0.017136
beta_concerns[4,2]  2.167e-01 1.0811 0.0038224       0.007361
beta_concerns[1,3] -4.606e+00 1.1887 0.0042027       0.013069
beta_concerns[2,3]  3.652e+00 1.2240 0.0043273       0.009667
beta_concerns[3,3] -6.413e-01 2.2112 0.0078177       0.019334
beta_concerns[4,3] -3.438e-02 1.0828 0.0038285       0.007581
beta_concerns[1,4] -4.719e+00 1.2266 0.0043368       0.015003
beta_concerns[2,4]  3.374e+00 1.2594 0.0044526       0.009576
beta_concerns[3,4] -6.152e-01 2.2043 0.0077935       0.018845
beta_concerns[4,4]  1.235e-01 1.0758 0.0038036       0.008539
beta_concerns[1,5] -3.860e+00 1.1347 0.0040118       0.012483
beta_concerns[2,5]  3.827e+00 1.2171 0.0043031       0.009305
beta_concerns[3,5] -4.915e-01 2.2007 0.0077808       0.018295
beta_concerns[4,5]  2.478e-02 1.0702 0.0037837       0.007021
beta_concerns[1,6] -4.767e+00 1.2245 0.0043293       0.014078
beta_concerns[2,6]  3.534e+00 1.2364 0.0043715       0.010239
beta_concerns[3,6] -6.452e-01 2.2097 0.0078123       0.019585
beta_concerns[4,6]  1.081e-01 1.0769 0.0038075       0.008460
beta_concerns[1,7] -4.069e+00 1.1367 0.0040187       0.012194
```

```
beta_concerns[2,7]   3.868e+00  1.2217 0.0043193        0.009820
beta_concerns[3,7]  -4.407e-01  2.2040 0.0077923        0.018519
beta_concerns[4,7]   1.545e-01  1.0699 0.0037826        0.007618
beta_concerns[1,8]  -3.940e+00  1.1366 0.0040187        0.012101
beta_concerns[2,8]   3.974e+00  1.2398 0.0043832        0.009477
beta_concerns[3,8]  -5.458e-01  2.1954 0.0077619        0.017095
beta_concerns[4,8]   1.167e-01  1.0742 0.0037979        0.007371
beta_concerns[1,9]  -4.337e+00  1.1564 0.0040884        0.013753
beta_concerns[2,9]   3.847e+00  1.2249 0.0043306        0.009322
beta_concerns[3,9]  -4.190e-01  2.2058 0.0077988        0.018446
beta_concerns[4,9]  -2.844e-03  1.0810 0.0038219        0.008959
beta_concerns[1,10] -4.073e+00  1.1363 0.0040174        0.012624
beta_concerns[2,10]  3.869e+00  1.2216 0.0043192        0.009701
beta_concerns[3,10] -5.698e-01  2.1984 0.0077725        0.017693
beta_concerns[4,10]  1.530e-01  1.0737 0.0037963        0.008289
beta_concerns[1,11] -4.881e+00  1.2552 0.0044379        0.014347
beta_concerns[2,11]  3.457e+00  1.2498 0.0044186        0.009917
beta_concerns[3,11] -6.286e-01  2.2055 0.0077975        0.019068
beta_concerns[4,11] -7.402e-02  1.0982 0.0038828        0.009194
beta_concerns[1,12] -4.845e+00  1.2666 0.0044781        0.016084
beta_concerns[2,12]  3.279e+00  1.2931 0.0045716        0.010467
beta_concerns[3,12] -3.806e-01  2.2162 0.0078355        0.019071
beta_concerns[4,12]  5.911e-02  1.0801 0.0038186        0.008641
beta_subjects[1,1]  -1.026e+00  1.7539 0.0062010        0.013963
beta_subjects[2,1]  -9.228e-03  0.9993 0.0035331        0.010402
beta_subjects[1,2]  -9.540e-01  1.7709 0.0062611        0.014246
beta_subjects[2,2]  -1.087e-02  1.0015 0.0035408        0.005352
beta_subjects[1,3]  -9.498e-01  1.7576 0.0062139        0.012297
beta_subjects[2,3]  -4.377e-02  1.0536 0.0037249        0.024890
beta_subjects[1,4]  -2.778e+00  1.6999 0.0060102        0.013600
beta_subjects[2,4]  -5.538e-01  1.1471 0.0040556        0.014060
beta_subjects[1,5]  -9.456e-01  1.7616 0.0062281        0.011485
beta_subjects[2,5]   2.732e-03  0.9895 0.0034983        0.006100
beta_subjects[1,6]  -9.741e-01  1.7918 0.0063351        0.012149
beta_subjects[2,6]   7.873e-03  1.0148 0.0035880        0.014768
beta_subjects[1,7]  -9.307e-01  1.7580 0.0062156        0.009516
beta_subjects[2,7]   2.486e-02  1.0248 0.0036232        0.019177
beta_subjects[1,8]  -1.011e+00  1.8066 0.0063874        0.019542
beta_subjects[2,8]  -1.169e-02  0.9837 0.0034779        0.005824
```

```
beta_subjects[1,9]  -2.657e+00  1.6955 0.0059946       0.015274
beta_subjects[2,9]  -5.435e-01  1.1334 0.0040072       0.013744
beta_subjects[1,10]  1.305e+00  1.3743 0.0048589       0.010574
beta_subjects[2,10]  2.365e-01  0.8442 0.0029848       0.006338
beta_subjects[1,11]  3.282e-01  1.2940 0.0045751       0.010909
beta_subjects[2,11]  5.100e-02  0.8097 0.0028627       0.009121
beta_subjects[1,12]  9.702e-02  1.0903 0.0038549       0.007760
beta_subjects[2,12]  3.882e-02  0.7457 0.0026364       0.004170
beta_subjects[1,13]  1.969e+00  1.4250 0.0050381       0.011695
beta_subjects[2,13]  2.880e-03  1.0025 0.0035442       0.007075
beta_subjects[1,14] -9.589e-01  1.7866 0.0063168       0.009318
beta_subjects[2,14] -2.490e-03  1.0241 0.0036209       0.013867
beta_subjects[1,15] -1.283e-01  1.1493 0.0040635       0.010010
beta_subjects[2,15] -3.461e-02  0.7862 0.0027797       0.013376
beta_subjects[1,16]  1.831e+00  1.3589 0.0048044       0.011410
beta_subjects[2,16]  3.356e-01  0.9037 0.0031950       0.014333
beta_subjects[1,17]  1.289e+00  1.5166 0.0053619       0.015653
beta_subjects[2,17]  2.967e-01  0.9284 0.0032824       0.007935
beta_subjects[1,18] -9.668e-01  1.7587 0.0062178       0.013757
beta_subjects[2,18]  1.050e-02  1.0016 0.0035413       0.010794
beta_subjects[1,19] -5.781e-01  1.1256 0.0039796       0.008881
beta_subjects[2,19] -1.123e-01  0.7751 0.0027405       0.007949
beta_subjects[1,20] -1.613e+00  1.3319 0.0047091       0.009314
beta_subjects[2,20] -3.344e-01  0.9141 0.0032319       0.009600
beta_subjects[1,21]  2.017e+00  1.3142 0.0046466       0.012362
beta_subjects[2,21] -1.237e-02  0.9993 0.0035331       0.007564
beta_subjects[1,22]  5.407e-01  1.1502 0.0040666       0.010489
beta_subjects[2,22]  8.505e-02  0.8033 0.0028400       0.014647
beta_subjects[1,23]  1.923e+00  1.3017 0.0046020       0.009320
beta_subjects[2,23]  1.239e-02  0.9888 0.0034961       0.009249
beta_subjects[1,24] -1.487e+00  1.3561 0.0047945       0.009297
beta_subjects[2,24] -3.027e-01  0.8928 0.0031564       0.009615
beta_subjects[1,25]  6.669e-01  1.1773 0.0041623       0.009053
beta_subjects[2,25]  1.343e-01  0.7777 0.0027498       0.004163
beta_subjects[1,26] -9.456e-01  1.7599 0.0062222       0.011556
beta_subjects[2,26]  6.327e-03  1.0332 0.0036528       0.016953
beta_subjects[1,27]  1.224e+00  1.2313 0.0043533       0.009189
beta_subjects[2,27]  2.385e-01  0.8353 0.0029531       0.007125
beta_subjects[1,28]  3.351e+00  1.3748 0.0048607       0.011238
```

```
beta_subjects[2,28]  6.563e-03  0.9817 0.0034709       0.005764
beta_subjects[1,29]  2.366e+00  1.4211 0.0050245       0.016062
beta_subjects[2,29]  4.862e-01  1.0463 0.0036993       0.017806
beta_subjects[1,30] -2.651e-01  1.1195 0.0039581       0.007968
beta_subjects[2,30] -4.654e-02  0.7618 0.0026933       0.008021
lp__                -2.003e+02 43.4391 0.1535805       1.314886

2. Quantiles for each variable:

                         2.5%       25%        50%        75%     97.5%
beta_ovall[1]        -6.80593  -4.99768 -4.247e+00  -3.59297   -2.4812
beta_ovall[2]         1.62036   2.88162  3.576e+00   4.36139    6.2391
beta_ovall[3]        -4.81769  -1.88661 -5.276e-01   0.83178    3.7715
beta_ovall[4]        -1.99255  -0.59057  7.928e-02   0.72437    2.0896
sigmac[1]             0.04015   0.36430  6.036e-01   0.84854    1.4359
sigmac[2]             0.01789   0.17224  3.776e-01   0.64676    1.2711
sigmac[3]             0.01454   0.14946  3.415e-01   0.66401    1.6597
sigmac[4]             0.01077   0.11100  2.432e-01   0.43971    1.0047
sigmas[1]             0.97641   1.65674  2.020e+00   2.44860    3.5374
sigmas[2]             0.01983   0.20677  5.112e-01   1.07388    2.4904
beta_concerns[1,1]   -7.00006  -5.09872 -4.305e+00  -3.60633   -2.4393
beta_concerns[2,1]    1.41708   2.78408  3.518e+00   4.33450    6.2641
beta_concerns[3,1]   -4.88128  -1.87557 -4.746e-01   0.92578    3.9600
beta_concerns[4,1]   -2.26195  -0.75316 -3.489e-02   0.64896    2.0420
beta_concerns[1,2]   -6.21239  -4.41738 -3.650e+00  -2.95032   -1.7022
beta_concerns[2,2]    1.61645   2.93424  3.672e+00   4.48012    6.4018
beta_concerns[3,2]   -4.90149  -1.92934 -5.274e-01   0.85797    3.8362
beta_concerns[4,2]   -1.93347  -0.47260  2.221e-01   0.90362    2.3708
beta_concerns[1,3]   -7.25636  -5.31688 -4.499e+00  -3.78159   -2.5670
beta_concerns[2,3]    1.46732   2.82429  3.572e+00   4.38709    6.3143
beta_concerns[3,3]   -5.09213  -2.04893 -6.340e-01   0.76747    3.7731
beta_concerns[4,3]   -2.22365  -0.72108 -1.296e-02   0.66512    2.0724
beta_concerns[1,4]   -7.42925  -5.45627 -4.614e+00  -3.85912   -2.6221
beta_concerns[2,4]    1.03812   2.53663  3.312e+00   4.14101    6.0680
beta_concerns[3,4]   -5.04775  -2.00347 -6.073e-01   0.78389    3.7831
beta_concerns[4,4]   -2.01817  -0.56247  1.360e-01   0.81172    2.2599
beta_concerns[1,5]   -6.36511  -4.53993 -3.776e+00  -3.08778   -1.8576
beta_concerns[2,5]    1.70214   2.99435  3.740e+00   4.56019    6.4977
beta_concerns[3,5]   -4.86763  -1.89809 -4.897e-01   0.91029    3.9043
```

```
beta_concerns[4,5]    -2.13384   -0.65380  3.933e-02    0.70959    2.1256
beta_concerns[1,6]    -7.47846   -5.50563 -4.659e+00   -3.91238   -2.6680
beta_concerns[2,6]     1.28786    2.71079  3.452e+00    4.27822    6.2000
beta_concerns[3,6]    -5.11199   -2.03983 -6.378e-01    0.77224    3.7426
beta_concerns[4,6]    -2.03829   -0.57534  1.195e-01    0.79912    2.2233
beta_concerns[1,7]    -6.59544   -4.74933 -3.977e+00   -3.29058   -2.0751
beta_concerns[2,7]     1.71912    3.03277  3.775e+00    4.60208    6.5519
beta_concerns[3,7]    -4.83512   -1.84355 -4.388e-01    0.94655    4.0071
beta_concerns[4,7]    -1.97232   -0.52830  1.583e-01    0.83899    2.2799
beta_concerns[1,8]    -6.43956   -4.62570 -3.850e+00   -3.15909   -1.9547
beta_concerns[2,8]     1.81171    3.12712  3.875e+00    4.71180    6.7143
beta_concerns[3,8]    -4.92164   -1.94687 -5.446e-01    0.85725    3.7922
beta_concerns[4,8]    -2.02920   -0.56394  1.260e-01    0.80094    2.2482
beta_concerns[1,9]    -6.90542   -5.02757 -4.242e+00   -3.53666   -2.3302
beta_concerns[2,9]     1.69801    3.01791  3.750e+00    4.58272    6.5460
beta_concerns[3,9]    -4.79982   -1.82417 -4.262e-01    0.97361    4.0158
beta_concerns[4,9]    -2.19872   -0.68690  2.097e-02    0.69271    2.0954
beta_concerns[1,10]   -6.58232   -4.75869 -3.978e+00   -3.28779   -2.0955
beta_concerns[2,10]    1.71432    3.03974  3.775e+00    4.60068    6.5387
beta_concerns[3,10]   -4.95836   -1.96863 -5.719e-01    0.83290    3.8482
beta_concerns[4,10]   -1.98839   -0.53441  1.609e-01    0.83558    2.2848
beta_concerns[1,11]   -7.66023   -5.64414 -4.768e+00   -3.99696   -2.7294
beta_concerns[2,11]    1.17131    2.61853  3.379e+00    4.21755    6.1351
beta_concerns[3,11]   -5.07293   -2.03175 -6.216e-01    0.76869    3.7531
beta_concerns[4,11]   -2.33464   -0.76662 -4.725e-02    0.63689    2.0394
beta_concerns[1,12]   -7.66754   -5.60000 -4.732e+00   -3.95485   -2.6863
beta_concerns[2,12]    0.85399    2.42907  3.219e+00    4.07411    6.0017
beta_concerns[3,12]   -4.78348   -1.79180 -3.968e-01    1.02647    4.0731
beta_concerns[4,12]   -2.10456   -0.62908  6.841e-02    0.75119    2.1911
beta_subjects[1,1]    -4.92575   -2.01946 -8.794e-01    0.12060    2.0965
beta_subjects[2,1]    -2.27622   -0.27983 -4.267e-05    0.27525    2.1291
beta_subjects[1,2]    -4.98906   -1.92771 -7.846e-01    0.21495    2.0981
beta_subjects[2,2]    -2.18735   -0.28740 -1.081e-03    0.26726    2.1458
beta_subjects[1,3]    -4.92511   -1.94706 -7.833e-01    0.21776    2.1110
beta_subjects[2,3]    -2.55748   -0.30358 -3.156e-03    0.26306    2.1478
beta_subjects[1,4]    -6.46854   -3.78386 -2.681e+00   -1.64790    0.2710
beta_subjects[2,4]    -3.77484   -0.82965 -1.462e-01    0.04234    0.8050
beta_subjects[1,5]    -4.89067   -1.93971 -7.872e-01    0.20435    2.1459
beta_subjects[2,5]    -2.12687   -0.27993 -8.030e-04    0.27402    2.1485
```

```
beta_subjects[1,6]    -4.94029   -1.99442 -8.348e-01    0.19982    2.1698
beta_subjects[2,6]    -2.20201   -0.28112 -1.137e-04    0.27931    2.3257
beta_subjects[1,7]    -4.81672   -1.96347 -7.755e-01    0.23234    2.1564
beta_subjects[2,7]    -2.13529   -0.27371  1.483e-04    0.28250    2.4714
beta_subjects[1,8]    -5.05119   -2.02400 -8.397e-01    0.16030    2.1819
beta_subjects[2,8]    -2.15423   -0.29186 -1.983e-03    0.26900    2.1437
beta_subjects[1,9]    -6.51149   -3.63316 -2.516e+00   -1.52350    0.2683
beta_subjects[2,9]    -3.70433   -0.82398 -1.396e-01    0.04559    0.8068
beta_subjects[1,10]   -1.33548    0.40871  1.265e+00    2.15831    4.1363
beta_subjects[2,10]   -1.20318   -0.12514  5.133e-02    0.47948    2.4241
beta_subjects[1,11]   -2.21522   -0.51056  3.086e-01    1.15555    2.9170
beta_subjects[2,11]   -1.62059   -0.23209  7.141e-03    0.30718    1.9187
beta_subjects[1,12]   -2.01837   -0.60712  7.905e-02    0.77861    2.3093
beta_subjects[2,12]   -1.55991   -0.22993  5.693e-03    0.29271    1.7636
beta_subjects[1,13]   -0.54669    0.99947  1.864e+00    2.81781    5.1065
beta_subjects[2,13]   -2.16908   -0.28306 -9.308e-04    0.27365    2.2282
beta_subjects[1,14]   -4.95401   -1.97051 -8.125e-01    0.19885    2.2270
beta_subjects[2,14]   -2.31600   -0.27548  2.745e-04    0.28028    2.2431
beta_subjects[1,15]   -2.39492   -0.85006 -1.425e-01    0.58929    2.1757
beta_subjects[2,15]   -1.89002   -0.28754 -4.737e-03    0.23952    1.6751
beta_subjects[1,16]   -0.80240    0.94289  1.816e+00    2.70624    4.5734
beta_subjects[2,16]   -1.03938   -0.08890  8.530e-02    0.59461    2.7564
beta_subjects[1,17]   -1.61141    0.29096  1.264e+00    2.24975    4.3782
beta_subjects[2,17]   -1.18965   -0.10946  6.678e-02    0.54255    2.8082
beta_subjects[1,18]   -4.87802   -1.95619 -8.193e-01    0.18009    2.1033
beta_subjects[2,18]   -2.13708   -0.27695  5.810e-04    0.27957    2.2885
beta_subjects[1,19]   -2.78238   -1.30092 -5.901e-01    0.13183    1.6719
beta_subjects[2,19]   -1.99713   -0.35936 -2.257e-02    0.18356    1.4602
beta_subjects[1,20]   -4.34007   -2.46190 -1.588e+00   -0.73552    0.9378
beta_subjects[2,20]   -2.83762   -0.58111 -8.047e-02    0.09246    1.0419
beta_subjects[1,21]   -0.34988    1.11314  1.941e+00    2.83003    4.8238
beta_subjects[2,21]   -2.20208   -0.28747 -1.412e-03    0.26838    2.1534
beta_subjects[1,22]   -1.73958   -0.19418  5.380e-01    1.27387    2.8371
beta_subjects[2,22]   -1.60144   -0.20448  1.498e-02    0.33867    1.9693
beta_subjects[1,23]   -0.42593    1.03562  1.851e+00    2.72658    4.7160
beta_subjects[2,23]   -2.14308   -0.26816  8.698e-04    0.28263    2.2289
beta_subjects[1,24]   -4.28578   -2.34415 -1.441e+00   -0.58815    1.0891
beta_subjects[2,24]   -2.68166   -0.55125 -7.092e-02    0.10127    1.1040
beta_subjects[1,25]   -1.62169   -0.07944  6.357e-01    1.39726    3.0697
```

```
beta_subjects[2,25]   -1.37080   -0.16896  2.719e-02    0.37217    2.0719
beta_subjects[1,26]   -4.87197   -1.94893 -7.753e-01    0.21500    2.1601
beta_subjects[2,26]   -2.20696   -0.27933  1.914e-04    0.28097    2.3825
beta_subjects[1,27]   -1.18425    0.41668  1.220e+00    2.01511    3.6699
beta_subjects[2,27]   -1.21114   -0.12188  5.460e-02    0.49261    2.3860
beta_subjects[1,28]    0.90990    2.40384  3.260e+00    4.19847    6.3514
beta_subjects[2,28]   -2.12256   -0.27598  8.721e-04    0.28258    2.1670
beta_subjects[1,29]   -0.41137    1.46176  2.342e+00    3.26053    5.2550
beta_subjects[2,29]   -0.85330   -0.05527  1.254e-01    0.76479    3.3894
beta_subjects[1,30]   -2.45136   -0.98824 -2.753e-01    0.44061    2.0114
beta_subjects[2,30]   -1.78566   -0.30474 -8.310e-03    0.22366    1.6297
lp__                -289.83603 -228.90350 -1.980e+02 -169.93025 -121.3489

> ##plot(fit.wind.mcmc, trace=FALSE, ask=TRUE)
```

Here, we summarize odds ratios—instead of log odds ratios—of overall concern effects (by merely exponentiating draws of effects from on log the odds scale to the odds scale. For example, $\exp(\beta_1)$ (`beta_ovall[2]` in the output, below) is the multiplicative change in the odds of overall health concern from those who do not use filtration to those who do use filtration. In other words, this is the odds ratio of filtration and is estimated to increase the odds of health concerns over 35 times (posterior median) for subject who use filtration compared to those subjects who do not use filtration.

```
> fit.wind.mcmc<- window(fit.mcmc[,names], start=10001)
> overallOR<- exp(as.array(fit.wind.mcmc[,1:4]))
> dim(overallOR)

[1] 20000     4     4

> overallOR.mcmc<- vector("list", nchains)
> (nchains<- nchain(fit.mcmc))

[1] 4

> for(chain in 1:nchains){
+     overallOR.mcmc[[chain]]<- coda::as.mcmc(overallOR[,,chain])
+     }
> overallOR.mcmc<- coda::as.mcmc.list(overallOR.mcmc)
> varnames(overallOR.mcmc)
```

```
[1] "beta_ovall[1]" "beta_ovall[2]" "beta_ovall[3]" "beta_ovall[4]"

> summary(overallOR.mcmc)


Iterations = 1:20000
Thinning interval = 1
Number of chains = 4
Sample size per chain = 20000

1. Empirical mean and standard deviation for each variable,
   plus standard error of the mean:

                  Mean        SD  Naive SE Time-series SE
beta_ovall[1]  0.02124   0.02286 8.082e-05      0.0003871
beta_ovall[2] 99.35379 658.02279 2.326e+00      3.1952869
beta_ovall[3] 13.23201 340.98050 1.206e+00      1.6971713
beta_ovall[4]  1.88364   6.58988 2.330e-02      0.0321392

2. Quantiles for each variable:

                  2.5%       25%      50%      75%     97.5%
beta_ovall[1] 0.001107  0.006754  0.01431  0.02752   0.08364
beta_ovall[2] 5.054908 17.843200 35.71372 78.36579 512.38165
beta_ovall[3] 0.008085  0.151585  0.59003  2.29741  43.44364
beta_ovall[4] 0.136347  0.554014  1.08251  2.06344   8.08168
```

As another example, the odds of health concern for a typical (44.5 yr old) male subject is about 0.51 (posterior median) ($\exp(\beta_0 + \beta_1)$).

```
> ## E.g., odds of filtration for middle aged (44.5 yrs) male subject (e.g.)
> tmp<- exp(unlist(fit.wind.mcmc[,1]) + unlist(fit.wind.mcmc[,2]))
> mean(tmp)

[1] 0.78129

> sd(tmp)

[1] 1.118238

> quantile(tmp,probs=c(0.025,0.25,0.5,0.75,0.975))
```

```
      2.5%        25%        50%        75%      97.5%
0.07884503 0.27920053 0.51099780 0.92182950 3.08450905
```
